# In Utero Stem Cell Transplantation: Potential Therapeutic Application for Muscle Diseases

**DOI:** 10.1155/2017/3027520

**Published:** 2017-05-15

**Authors:** Neeladri Chowdhury, Atsushi Asakura

**Affiliations:** ^1^Stem Cell Institute, University of Minnesota Medical School, Minneapolis, MN 55455, USA; ^2^Paul and Sheila Wellstone Muscular Dystrophy Center, University of Minnesota Medical School, Minneapolis, MN 55455, USA; ^3^Department of Neurology, University of Minnesota Medical School, Minneapolis, MN 55455, USA

## Abstract

Muscular dystrophies, myopathies, and traumatic muscle injury and loss encompass a large group of conditions that currently have no cure. Myoblast transplantations have been investigated as potential cures for these conditions for decades. However, current techniques lack the ability to generate cell numbers required to produce any therapeutic benefit. In utero stem cell transplantation into embryos has been studied for many years mainly in the context of hematopoietic cells and has shown to have experimental advantages and therapeutic applications. Moreover, patient-derived cells can be used for experimental transplantation into nonhuman animal embryos via in utero injection as the immune response is absent at such early stages of development. We therefore propose in utero transplantation as a potential method to generate patient-derived humanized skeletal muscle as well as muscle stem cells in animals for therapeutic purposes as well as patient-specific drug screening.

## 1. Introduction

Skeletal muscle is the most abundant tissue in the human body, comprising 40–50% of body mass and playing vital roles in locomotion, heat production, and overall metabolism. Loss of muscle is a serious consequence of many chronic diseases including muscular diseases such as Duchenne muscular dystrophy (DMD) and aging-related sarcopenia because it leads to muscle weakness, loss of independence, and increased risk of death. In addition, traumatic muscle injury and loss caused by accident, surgery, and wartime injuries needs prolonged recovery.

Muscular dystrophies are a large and diverse group of genetic disorders that are associated with progressive loss of muscle mass and strength. The most common forms, DMD and Becker muscular dystrophy (BMD), are a result of mutations of the *DMD* gene on the X chromosome that code for the large sarcolemmal protein dystrophin. The rate of occurrence of DMD is reported to be in between 1 : 3802 and 1 : 6291 male births [1] and that of BMD is about 1 : 18,450 male births [2]. DMD is a more severe form and is caused by a complete absence of the dystrophin protein, whereas BMD is a milder form associated with lower levels of expression of dystrophin or a truncated dystrophin protein. DMD patients experience a loss of ambulation and are normally wheelchair dependent by 12 years of age followed by cardiac and respiratory failure in the second decade of life that are the main causes of death [3]. The dystrophin protein is one of the largest proteins produced in the human body containing several distinct domains. The N-terminus sequences are highly homologous to actin-binding domain responsible for interaction with the cytoskeleton. The central region consists of 24 rod-shaped spectrin-like repeats made up of triple helices. Each repeat is separated by nonhelical regions called hinges. The C-terminus region shows homology with utrophin and is responsible for binding and interacting with multiprotein dystrophin-associated protein (DAP) complex and the extracellular matrix (ECM) [4]. The large size and multiple domains of the dystrophin protein signify that it is capable of binding to multiple proteins and may perform a variety of functions. A common belief is that it acts as a spring that disperses the forces experienced by the sarcolemma during muscle contractions and prevents membrane damage [5, 6]. The lack of dystrophin in DMD prevents this force dispersion causing excessive damage to the sarcolemma which is responsible for the progressive degeneration of the muscle fibers with age. While the skeletal muscle possesses a tremendous capacity for regeneration, this potential ultimately declines with DMD. No treatments are currently available for DMD, terminal muscle diseases.

Most organs in the body contain a population of tissue-resident stem cells that are able to proliferate and differentiate to repair the organs in the case of damage while undergoing self-renewal to maintain a constant pool of stem cells. In the skeletal muscle, this cell population is known as satellite cells due to their anatomic location between the myofiber and the basal lamina [7]. They proliferate in response to damage to give rise to muscle progenitor cells or myoblasts that then fuse to existing muscle fibers to repair the damage or give rise to new fibers [8], while myoblasts also possess adipogenic and osteogenic differentiation potential in vitro [9]. Apart from satellite cells, many atypical cell types such as side population cells, neural stem cells, hematopoietic stem cells, mesoangioblasts, pericytes, CD133+ circulating cells, and mesenchymal stem cells (MSCs) have been shown to possess myogenic differentiation potential [10–15]. One of the most promising uses for stem cells is the possibility to treat muscle diseases including those that have their origins in genetic anomalies and traumatic muscle injury and loss caused by accident, surgery, and wartime injuries.

## 2. Myoblast Transplantation for DMD Therapy

Due to the highly proliferative capacity of satellite cells, their transplantation has been investigated for the treatment of muscular dystrophies. In some of the earliest myoblast transplantation studies performed by Partridge in the late 1980s, they transplanted mononuclear cells isolated by disaggregation of normal neonatal muscle into nude, phosphorylase kinase- (PhK-) deficient mice. Upon harvesting the muscles and checking for PhK expression, they found that the transplanted cells contributed to the formation of new myofibers as well as fused to existing myofibers enabling them to express PhK. Different isoenzymes of glucose-6-phosphate isomerase (GPI) in donor versus recipient muscle were used to determine the animal of origin [16]. Similar experiments performed in the *mdx* mouse model for DMD showed dystrophin-positive fibers in injected muscle. Interestingly, they observed higher levels of engraftment compared to the previous study, indicating that actively regenerating muscle may be important for better engraftment of transplanted cells [17]. Experiments performed by Morgan et al. showed long-term engraftment and regenerative capacity of transplanted myoblasts. Their experiments showed better engraftment in irradiated muscle when compared to nonirradiated contralateral controls [18]. Furthermore, myoblast transplantation performed in nonhuman primates using an immunosuppressive agent (tacrolimus) showed significant levels of survival and engraftment of transplanted cells when compared to control [19]. However, the prolonged use of tacrolimus is toxic, and therefore, to reduce the effective dosage, Skuk et al. combined it with mycophenolate mofetil (MMF), another immunosuppressive, and observed fewer levels of serum antibodies and CD8+ T cells at the sites of injection in their nonhuman primate experiments [20].

The above experiments taken together provide compelling evidence for the potential of myoblast transplantation as a therapeutic technique for the treatment of muscular dystrophies in humans. These inspired multiple clinical trials in the early 90s that did not prove to be very successful due to insufficient amounts of research done in preclinical trials to determine the best protocol for myoblast transplantation [21]. Among the many reasons for the failure of the trials were the large amounts of cell death observed in the transplanted myoblasts as well as immune reactions against the donor myoblasts and fibers that were previously thought to not express the MHC class II. Furthermore, the limited migration of the transplanted myoblasts from the sites of transplantation added to the inefficiency of the procedure [22].

## 3. iPSC-Derived Myogenic Cells for Muscular Dystrophy Therapy

**Figure 1:**
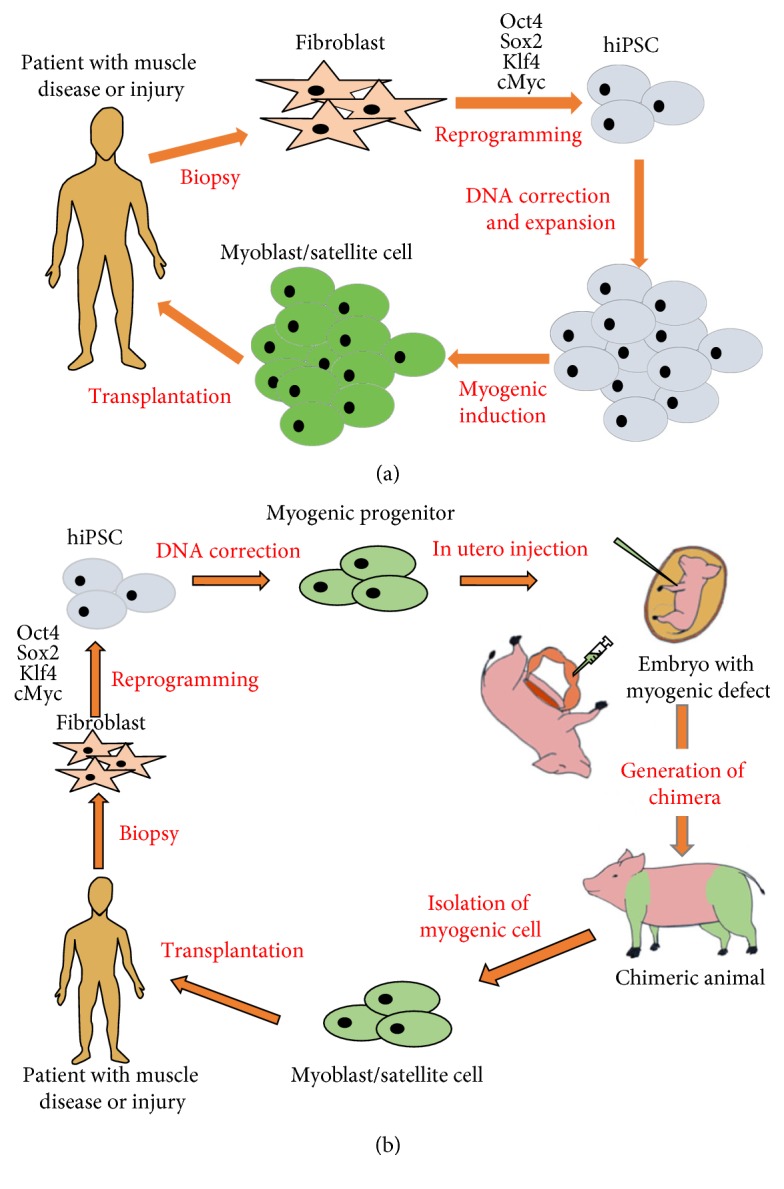
Current and new approaches for iPSC-derived stem cell transplantation for muscle diseases. (a) Patient-derived skin fibroblasts will be reprogramed into iPSCs by reprogramming factors. Patient-derived iPSCs will be used for DNA correction of dystrophin mutation by DNA-editing technologies. These corrected iPSCs will be induced to myogenic differentiation to generate myogenic progenitor cells which will be used for autologous cell therapy for patients suffering from muscle diseases and traumatic muscle injury and loss. (b) Patient-derived skin fibroblasts will be reprogramed into iPSCs by reprogramming factors. Patient-derived iPSCs will be used for DNA correction of dystrophin mutation by DNA-editing technologies. These corrected iPSCs will be used for myogenic progenitor cell induction followed by in utero injection into animal embryos carrying a defect of myogenic master genes such as *MyoD*, *Myf5*, and *MRF4*, allowing chimeric animal to develop human skeletal muscle. Chimeric animal-derived patient-specific myoblasts or satellite cells will be used for autologous cell therapy for muscle diseases and traumatic muscle injury and loss.

Recently, pluripotent stem cells have been investigated as sources of muscle progenitor cells for therapy due to their ability to differentiate into all three germ layers as well as their ease of expansion. The discovery of induced pluripotent stem cells (iPSCs), which enable the conversion of somatic cells to pluripotent cells by the introduction of a specific transcription factors, makes it possible to generate patient-specific stem cells, thus bypassing complications associated with immune rejection in case of transplants. Additionally, the iPSCs can be genetically corrected before transplantation, thus providing long-term cures for conditions like muscular dystrophies [15]. To overcome the problem of immune reactions and the large quantities of cells required to observe therapeutic benefits in large muscles, the use of autologous patient-derived iPSCs that can be proliferated indefinitely can be used for transplantation (Figure 1(a)). There are multiple methods including utilizing forced expression of myogenic transcription factors such as Pax3, Pax7, and MyoD and step-by-step induction methods which recapitulate embryonic myogenesis [23]. These protocols have been investigated to derive a variety of cell types having myogenic potential that has been reviewed by Darabi and Perlingeiro [24]. They identified some of the major hurdles to the use of iPSCs in therapeutic applications, such as the heavy dependence on gene overexpression to derive the myogenic precursors and the safety concerns associated with the use of these cells. However, efficient myogenic differentiation and the scale-up of myogenic differentiation remain elusive and must be developed further in order to generate effective cellular therapies. In addition, in vitro-induced myogenic cells from pluripotent stem cells only show embryonic muscle phenotypes but not mature muscle phenotype [23], limiting the use of iPSC-derived myogenic cells for clinical situation. It is therefore essential to develop alternative approaches to induce and obtain large numbers of satellite cells if the potential of myoblast transplantation as a therapeutic method is to be realized.

## 4. In Utero Stem Cell Transplantation (Table 1)

In utero transplantation (IUT) is based on the idea that the introduction of donor cells into a fetus at an early stage of development can result in the development of chimerism without the risk of rejection of the donor cells due to the undeveloped fetal immune system. The first evidence for this came in 1945 with Owen's observations on the blood types of bovine twins [25]. In his observations, Owen noticed that when a twin sire mated, it failed to transmit some of the antigens present on its own blood cells in any of his twenty progenies. Examination of the antigens present in his twin pointed to the possibility that these antigens could be derived from the twin. In a second observation of a case of superfecundation, he noticed that the twins possessed two antigens each that could not have been inherited from their respective sires or the dam but could have been obtained from the cosire. These observations led him to conclude that the cells containing these antigens were derived from a subset of cells that were interchanged during early embryonic development and were able to give rise to these erythrocytes throughout their lives. We now know these cells to be hematopoietic stem cells (HSCs).

Following these observations, a series of experiments were performed by Billingham and colleagues in which they grafted a variety of skin combinations in between monozygotic and dizygotic twins with the aim to use this as a method to distinguish between the two types of twins [26, 27]. Their experiments were unable to find a significant difference in response to homografts between dizygotic and monozygotic twins, and they also observed varying levels of tolerance to the homografts in the dizygotic twins. These results further bolstered evidence for acquired for tolerance towards the homograft during early embryonic development. The final study that showed that exposure to antigens early in fetal development could result in the acquisition of tolerance towards those antigens was performed by Billingham et al. Here, they injected tissue homogenates from the spleen, testes, and kidneys of adult mice from A-line mice into 6 embryos of CBA mice. Five embryos survived and were grafted with the skin of A-line mice at eight weeks of age. Two of the five mice showed complete tolerance of the graft, whereas one mouse showed prolonged tolerance followed by rejection after 90 days. The two mice that showed complete tolerance were then injected with lymph nodes of CBA mice that were previously immunized against A-strain mice which resulted in rapid rejection of the previously tolerated skin grafts showing that the tolerance was due to tolerance of the graft by the mice and not due to antigenic modifications by the graft [28].

With this evidence in mind, a number of experiments were carried out to demonstrate the feasibility of IUT for therapeutic purposes. Hematopoietic stem cells (HSCs) being easy to isolate and the most well-studied stem cells were used most commonly in these studies. Moreover, tests for engraftment of HSC could be easily performed through blood draws and biopsies of the spleen, liver, and thymus to check for progeny of the transplanted cells. The first attempt to show the feasibility of IUT of HSCs (IUT-HSCs) was conducted by Fleischman and Mintz [29] who utilized W/W mice that are lethally anemic as well as *W^v^*/*W^v^* mice that are viably anemic. They injected C57BL/6 fetal liver HSCs into the W/W mice and DBA/2 fetal liver HSC into the *W^v^/W^v^* mice at gestational day 11. Blood tested at different time points for the type of hemoglobin in the RBCs that showed most of the RBCs were of donor origin indicating successful engraftment of the fetal liver HSCs. Engraftment of in utero-transplanted cells into normal mice was also shown in another study using PCR to detect donor cells, which proved to be a more sensitive assay [30]. They were also able to transplant allogeneic skin grafts onto the chimeric mice and observe varying degrees of tolerance towards the grafts. Further studies have shown that the mode of injection [31] is also important for improving engraftment in IUT-HSCs. IUT-HSCs have also been shown to be successful in dogs [32], sheep [33], and monkeys [34, 35]. Following these studies, IUT-HSCs have been used in a number of experimental treatments in humans to treat diseases like SCID [36–39] and bare lymphocyte syndrome [40].

IUT has also been shown to be successful for other cell types. Human MSCs have also been shown to successfully engraft in multiple tissues in sheep following in utero intraperitoneal transplantation and could be detected over a period of 13 months [41]. Similar results have been shown for fetal liver-derived MSCs in sheep and human placenta-derived MSCs in rats [42, 43]. Due to the multipotentiality of MSCs, they can possibly be used to treat a variety of conditions. In rats, MSCs injected into the spinal cord of fetuses, which were induced to have spina bifida by the administration of retinoic acid, engrafted and expressed markers of motor neurons, neurons, sensory neurons, and neural precursor cells while inducing the expression of neurotrophic factors from the surrounding tissue [44, 45]. Transplanted MSCs also showed improved bone mineralization in mouse models of osteogenesis imperfecta and could be detected in a human patient suffering from the same disease after transplantation, indicating their potential as a possible therapeutic avenue for osteogenesis imperfecta [46, 47]. Engraftment post IUT has also been shown for amniotic fluid-derived cells [48, 49] and hepatocytes [50, 51].

A recent study conducted by Cohen et al. [52] further added to the growing body of studies proving the feasibility of IUT of human stem cells. In their study, they utilized primary mouse neural crest cells (NCCs) obtained from E8.5 GFP expressing embryos from C57BL/6 background and injected these cells into a nonpigmented *W^sh^/W^sh^ c*-*Kit* mutant mouse lacking endogenous melanoblasts. They then examined the coats of postnatal mice for pigmentation which would arise only from the donor cells, which was confirmed by checking for GFP. They were also able to obtain similar results from mouse ESC-derived NCCs and rat iPSC-derived NCCs. To prove that human cells could obtain similar levels of chimerism, they used hESC-derived NCCs and hiPSC-derived cells from an African American donor that were transfected with GFP. They examined the mice between E10.5 and E13.5 as well as postnatally for human chimerism and using immunohistochemistry, microscopy, and qPCR for analyzing human mitochondrial DNA. They obtained around 35% human chimerism at lower efficiencies than the mouse-mouse chimeras. However, this study highlights the potential for generating human tissue in animal models that can then be used as a model to study disease development, used to determine a cure for the condition, or used as a source of cells/tissue for transplantation.

## 5. Potential for Use of IUT in Treatment of Muscle Diseases

Prenatal diagnosis allows for the detection of genetic diseases early in gestation. While parental genetic screening and testing to identify carriers of mutations that can cause myopathies followed by in vitro fertilization (IVF) and preimplantation genetic screening could prevent many cases of BMD or DMD from occurring, these will only lead to a reduction and not an eradication of the disease since a third of DMD cases occur due to de novo mutations and cannot be preemptively screened for [53]. To combat this prenatal screening of fetuses could provide an avenue to identify fetuses that carry mutations that can cause BMD or DMD. Traditionally, this is done through chorionic villus sampling (CVS) and amniotic fluid sampling which are invasive procedures and pose a 0.5% to 1% risk of embryonic death [54]. Moreover, these procedures often involve ex vivo culturing of the cells isolated to get a sufficient amount of DNA to be tested which can introduce variabilities and culture-associated abnormalities. Recent advances in these technologies however have made it easier to detect these diseases with lower risks of mortality. For example, the discovery of cell-free fetal DNA (cffDNA) present in maternal plasma enabled new noninvasive techniques of detection to be researched. Using this cffDNA and coupling it with relative haplotype dosage analysis (RHDA), Parks et al. have been able to accurately predict the occurrence of DMD and BMD [54]. In spite of the limitation of using cffDNA in the case of twins, or if the mother has been the recipient of transplants, the technique is a step forward in enabling earlier diagnoses of congenital diseases which can then be coupled with in utero interventions to cure the condition.

Naturally, the logical step following early detection of a disease-causing mutation is the early remediation of the mutation. To this end, many groups have investigated the potential of in utero gene transfer or in utero gene correction as a potential method to treat monogenic myopathies. VSV-G-, Mokola-, and Ebola-pseudotyped lentiviral vectors, adenoviral vectors, and adeno-associated viral vectors have been shown to be highly efficient in targeting cardiac muscle and skeletal muscle including satellite cells following intramuscular or intraperitoneal injections in utero [55–57]. Utilizing an equine infectious anemia virus (EIAV) of the VSV-G pseudotype, the *β-galactosidase* (*lacZ*) gene was successfully delivered to most of the respiratory muscles and limb skeletal muscle via combined intrathoracic, intraperitoneal, and intramuscular injections, and notably, no immune responses were detected towards the viral proteins for up to 5 months of age [58]. To prevent the chances of deleterious mutations due to nonspecific integration of the transgene into the genome, nonintegrating viral vectors may be a better option. The delivery of the HC-Ad adenovirus that has a large insert capacity into the muscles of E16 mouse limbs showed stable expression of lacZ up to 5 months of age and was also able to successfully deliver dystrophin cDNA and restore dystroglycan complex expression in the limbs of *mdx* mice; however, functional recovery was meagre [59, 60]. Similar results were observed utilizing AAV8 vectors carrying the minidystrophin gene [61, 62]. For a large animal model, a protocol for in utero ultrasound-guided adenoviral vector delivery to the sheep fetal muscle has been published for skeletal muscle repair. Finally, in utero delivery of oligodeoxynucleotides into *mdx* mice has been examined for dystrophin gene correction [63].

Following the success of IUT-HSCs, many groups have tried to replicate similar successes for myogenic tissue repairs. Multiple cell types have been utilized for these studies. Liechty et al. were able to show successful engraftment of normal human MSCs following in utero intraperitoneal transplantation into fetal sheep. They observed human cells in multiple organs including skeletal muscle and cardiac tissue [41, 64, 65]. Mackenzie et al. also transplanted bone marrow (BM) cells and fetal liver cells isolated from *Rosa26* donor mice (transgenic for *lacZ*) into *mdx* mouse embryos (E14) and characterized their chimerism and engraftment at 4 weeks after birth. After determining hematopoietic chimerism, they discovered the presence of donor derived myogenic cells in the diaphragm, cardiac, and skeletal muscles of the chimeric mice but were unable to show dystrophin expression due to the low levels of engraftment [66]. Utilizing a more primitive group of cell type isolated from the somites of E11.5 mice and a less invasive procedure of injection into the uterine continuation of medial circumflex femoral veins of *mdx* mice, Torrente et al. were able to show the restoration of dystrophin expression in various skeletal muscles [67]. Surprisingly, the transplanted cells were able to cross the placenta and migrate to the sites of myogenesis. More recently, human fetal MSCs have been shown to successfully differentiate into cardiac and skeletal muscle following IUT into *mdx* mice [68]. In this study, different routes of cell transplantation (intramuscular, intraperitoneal, and intravascular) were compared, and the authors identified that intraperitoneal injection allows for the most widespread distribution of the cells while intravascular injection led to complete mortality of the embryos. Intramuscular injection resulted in more localized engraftment and reduced differentiation of the cells. Although this method was not tested in the *mdx* mice, the limitation might be overcome by matching the transplanted cells to the developmental stage of the embryos.

Although IUT may not be an ideal method in the case of muscular dystrophies and myopathies due to the complexity of myogenesis and the enormity of the tissue, it could be used to generate an unlimited source of myoblasts for transplantation into patients, addressing one of the main limitations to myoblast transplantation previously discussed in this paper. The generation of humanized organs in a host animal is a potential approach for regenerative medicine to repair muscle in patients suffering from myopathic diseases. For the creation of humanized organs in animals, it is essential to selectively knock out genes in the blastocysts that are critical for organ development [69]. *MRFs*, *Pax3*, or *Lbx1* mutant mice provide an ideal model since mice carrying the gene mutation(s) display a complete absence of muscle as a whole or at the level of the limb, respectively [70–72], supplying the empty niche for myogenesis. However, since injection of human stem cells into pregastrulation embryos has an ethical issue [73], IUT of stem cells into genetically modified mouse embryos is a potential approach for generating humanized organs. The clinical significance of this approach is the production of humanized muscle using specific gene mutant mouse embryos via IUT of iPSCs, which are developmentally vacant of the limb muscle. These humanized organs created in mice will serve as an animal model to study human muscle diseases and responses to pharmacological agents. In addition, muscle engineered in these strategies holds potential as a source for muscle stem cell transplantation for patients suffering from myopathic diseases. Therefore, they can be used as a platform to develop IUT for the purposes of generating human limb muscle in these mice. Translation to large animal models following these studies can result in the generation of patient-specific myoblasts that can then be harvested and transplanted as a possible therapeutic option (Figure 1(b)). Our preliminary results in normal mice show that transplanted myoblasts or iPSC-derived myogenic cells can survive in the developing embryo post IUT; however, their contribution to myogenesis is currently undetermined. If successful, IUT of gene-corrected iPS-derived precursors into growing fetuses of animals like pigs, sheep, or goat can be used to generate patient-specific muscle for a source of autologous myoblast transplantation.

The generation of patient-specific myogenic cells in host animals can be directly used for stem cell-based therapeutic transplantation in DMD and myopathic diseases. In addition, we can develop personalized muscle model carrying individual disease-associated mutations in the humanized animals. Potentially, such insights and developments will lead to new therapeutic interventions for myopathic diseases including DMD. With respect to expected outcomes, the work proposed in the aims of this study is collectively expected to provide new therapeutic interventions that will aid the growing number of people in this country who suffer from muscle degenerative diseases and traumatic muscle injury and loss. In addition, it is expected that the results will fundamentally advance the fields of muscle regeneration and stem cell biology.

## Figures and Tables

**Table 1:** In utero cell transplantation.

Author	Year	Ref	Host animal	Donor animal	Cell type	Target tissue	Injection site	Injection stage	Disease	Duration	Number of cells	Chimerism
Fleischman et al.	1979	[29]	W/W, *W^v^/W^v^* mouse	C57Bl/6, DBA/2 mouse	Fetal liver cell (E13–E15)	Hematopoiesis	Intraplacental	E11	No	—	1 × 10^5^	In peripheral blood is more in W/W than in *W^v^/W^v^*
Carrier et al.	1995	[30]	BALB/c, C57Bl/6 mouse	C57Bl/6 mouse	Fetal liver cell	Hematopoiesis	IP, intraplacental	E11–13	No	22–44 weeks	5 × 10^5^	10–62% (peripheral blood, liver, and spleen)
Archer et al.	1997	[74]	Nod/Scid, C57Bl/6 mouse	C57Bl/6-Ly-5.2 mouse	BM (lin-depleted)	Hematopoiesis	IP	E13.5	No	PB analyzed 4 and 26 weeks	8 × 10^5^	~55% (peripheral blood, spleen, BM)
Kim et al.	1999	[75]	BALB/c mouse	DBA/2 mouse	BM	Hematopoiesis	IP	E14	No	~8 weeks	—	Successful skin grafts in 2 of 3 mice
Mackenzie et al.	2002	[66]	mdx C57Bl/10 mouse	Rosa26 mouse	BM, fetal liver cell	Multiple	IP	E14	DMD	~14 months	5 × 10^6^ BM, 1 × 10^6^ fetal liver cell	Multiple tissues
Chou et al.	2005	[76]	BALB/c mouse	Human	MSC (BM)	Multiple	IP	E13-14	No	~5 months (postnatal)	1 × 10^5^	~56% in multiple tissues
Frattini et al.	2005	[77]	Oc+/−C57Bl/6 mouse	Mouse CD1 CMV-GFP	BM	Hematopoiesis	IP	E14.5	Autosomal recessive osteopetrosis	~7 months	5 × 10^6^	Improved survival
Chan et al.	2007	[68]	MF1 and mdx C57Bl/10 mice	Human	MSC (fetal)	Muscle	IP, yolk sac vein, intramuscular	E14–16	DMD	~18 weeks (postnatal)	5 × 10^3^–1 × 10^6^	Multiple tissues
Li et al.	2007	[78]	oim/oim mouse	Mouse	MSC (BM)	Multiple	IP	—	No	—	—	Multiple tissues
Guillot et al.	2008	[79]	oim/oim mouse	Human	MSC (fetal)	Bone	IP	E13.5–15	Osteogenesis imperfecta	E18, 1 week, 2 weeks, 4 weeks, 8 weeks, and 12 weeks	—	Multiple tissues
Kun-Yi Lin et al.	2013	[80]	Mouse	Mouse	Amniotic fluid progenitor cells	Multiple	IP	E13.5	No	3, 6, and 9 weeks (postnatal)	—	Multiple tissues
Ihara et al.	2015	[81]	MPSVII mouse	ICR/B6 actin-GFP mouse	BM (lin-depleted)	Hematopoiesis	IV in vitelline vein	E14.5	Mucopolysaccharidosis type VII	~8 weeks (postnatal)	5 × 10^5^	Multiple tissues
Cohen et al.	2016	[52]	Mouse (*W^sh^/W^sh^*)	Mouse, rat, and human	Mouse neural crest cell, mESC, riPSC, and hESC/hiPSC	Melanocyte	Intra-amniotic	E8.5	No	E10.5–E18.5, postnatal	—	Skin pigmentation
Boelig et al.	2016	[31]	C57Bl/6TgN mouse	C57Bl/6 H-2K actin-GFP mouse	BM	Hematopoiesis	IV in vitelline vein, IP, intrahepatic	E14	No	4, 24, 72 hrs, and ~6 months (postnatal)	5 × 10^6^ IV, IP, intrahepatic, 2 × 10^7^ IV	Multiple tissues
Munoz-Elias et al.	2004	[82]	Rat	Rat	MSC (BM)	Brain	Lateral ventricle	E15.5	No	E17.5, E19.5, and E21.5; 3 days , 1 month, and 2 months (postnatal)	—	Neuronal tissues
Chen et al.	2009	[43]	Rat	Human	MSC (placental)	Multiple	IP	E17	No	E21, 3 weeks, 12 weeks (postnatal)	—	~60% in multiple tissues
Li et al.	2012	[83]	Wistar rat	Wistar rat	BM-MSC	Neurogenesis	Lumbosacral spine	E16–18	Spina bifida aperta	E20	1–6 × 10^3^	Neuronal tissues
Munoz-Saez et al.	2013	[51]	Wistar rat	Wistar rat, Fischer	Fetal hepatocyte (E21)	Liver	IP	E17	No	~15 days (postnatal)	1 × 10^6^	Multiple tissues
Li et al.	2014	[44]	Rat	Rat	MSC (BM)	Spinal cord	Spinal column	E16	Spina bifida aperta	~E20	—	Spinal cord
Burai et al.	2015	[84]	Rat	Human	Amniotic cell	Multiple	—	E18	No	1, 4, 11, and 18 days	2 × 10^5^	Multiple tissues
Touraine et al.	1989	[40]	Human	Human	Fetal liver, thymic epithelial cell	Hematopoiesis	Umbilical vein	30 weeks	Bare lymphocyte syndrome	—	1.6 × 10^7^	10% in lymphocyte
Wengler et al.	1996	[37]	Human	Human	T cell-depleted CD34+ BM	Hematopoiesis	IP	21 weeks	No	—	—	No GVHD, normal T cell response
Flake et al.	1996	[36]	Human	Paternal	BM (CD34+ paternal)	Hematopoiesis	IP	16–18.5 weeks	—	At birth, 3 months, 6 months (postnatal)	1.48–2 × 10^6^	All T cells
Gil et al.	1999	[38]	Human	Human	BM (paternal CD34+)	Hematopoiesis	IP	23 weeks and 23 weeks + 10 days	SCID	—	1.4 × 10^7^, 6 × 10^6^	T cells
Westgren et al.	2002	[39]	Human	Human	Fetal liver cell (10 weeks)	Hematopoiesis	IP	14 weeks	X-linked SCID	—	7 × 10^7^	10% (24 weeks), 50% (33 weeks), all T cells/NK cells are donor origin
Le Blanc et al.	2005	[46]	Human	Human	MSC (fetal liver)	Bone	Umbilical vein	32 weeks	Osteogenesis imperfecta	—	6.5 × 10^6^	Normal osteoblast distribution and trabeculae
Götherström et al.	2014	[85]	Human	Human	MSC (7-week, 3-day/10-week fetal liver)	Liver	Intrahepatic vein	31 weeks	Osteogenesis imperfecta	3–10 years (postnatal)	4 × 10^7^	7.4%, healed fractures
Harrison et al.	1989	[35]	Rh monkey	Rh Monkey	Fetal liver cell (days 59–68 opposite sex)	Hematopoiesis	IP	60–62 days	No	~2 years	1 × 10^8^–1 × 10^9^/kg	Lymphoid, myeloid, and erythroid, no GVHD
Mychaliska et al.	1997	[34]	Monkey	Paternal	BM (T cell depleted)	Hematopoiesis	IP	61 days	No	~2 years	1 × 10^8^/kg	<0.1%, slower progression of rejection after kidney transplants
Shields et al.	2003	[86]	Baboon/cynomolgus monkey	Baboon monkey	BM (parental, T cell-depleted B), T cell	Hematopoiesis	IP	0.34–0.38 gestation	No	~2 years	3 × 10^9^	Peripheral blood
Asano et al.	2003	[87]	Cynomolgus monkey	Cynomolgus monkey	ESC	Multiple	IP or intrahepatic	End of 1st trimester	No	1, 3 months (posttransplantation)	3.6–4.8 × 10^6^	Multiple tissues, small tumor
Crombleholme et al.	1990	[88]	Lamb	Sheep	BM (whole, T cell depleted)	Hematopoiesis	IP	—	No	—	—	18% (whole), 6% (T cell depleted), No GVHD
Zanjani et al.	1994	[89]	Sheep	Human	CD45+ cell injected with human fetal HSC	Hematopoiesis	IP	50–54 days	No	~68 weeks (postnatal)	4.9 × 10^6^	Chimerism found in 2 of 6 sheep
Almeida-porada et al.	1999, 2000	[90, 91]	Sheep	Human	BM + stromal cell	Hematopoiesis	IP	55–60 days	No	3, 6, and 9 weeks after transplantation; 3 days, 3 months, and 1–3 years (postnatal)	5 × 10^4^–7.5 × 10^5^	Peripheral blood
Liechty et al.	2000	[41]	Sheep	Human	MSC (BM)	Multiple	IP	65 days, 85 days	No	2 weeks, 2 months, 5 months, and 13 months	—	Multiple tissues
Mackenzie et al.	2001	[66]	Sheep	Human	MSC	Multiple	IP	65 days, 85 days	No	—	2 × 10^6^	Multiple tissues in 28 of 29 sheep
Emmert et al.	2013	[92]	Sheep	Human	MSC (adipose/BM)	Multiple	IP and intramyocardial	70–75 days	No	7–9 days (postnatal)	—	Multiple tissues
Jeanblanc et al.	2014	[93]	Sheep	Sheep/human (opposite sex)	BM (T cell depleted), BM (CD34+), and BM (human CD34+)	Hematopoiesis	IP	45 days, 65 days	No	10, 60, and 130 days and ~9 months (posttransplantation)	5 × 10^5^ BM, 1.4 × 10^6^ CD34+, and 4 × 10^4^–5 × 10^5^ human CD34+	In blood cells at day 65
